# Dental implants rehabilitation in a patient with head and neck radiotherapy for osteosarcoma in the jaw. A clinical case report

**DOI:** 10.4317/jced.57863

**Published:** 2021-04-01

**Authors:** Pablo Garrido-Martínez, Juan-Francisco Peña-Cardelles, José-Juan Pozo-Kreilinger, Germán Esparza-Gómez, Néstor Montesdeoca-García, José-Luis Cebrián-Carretero

**Affiliations:** 1 DDS, phD. Associate Professor, Department of Prosthesis, Faculty of Dentistry, University Alfonso X el Sabio, Madrid. Department of Oral and Maxillofacial Surgery, Hospital La Luz, Madrid; 2 DDS. Professor of the Postgraduate Program in Oral Surgery and Implantology. Universidad Rey Juan Carlos, Madrid, Spain; 3 MD, DDS, phD. Associate Professor of Medicine. Department of Pathology. Universidad Autónoma de Madrid, Madrid. Hospital Universitario La Paz, Madrid; 4 MD, DDS, phD. Professor Titular, Faculty of Odontology, Universidad Complutense de Madrid, Madrid; 5 DMD, phD. Chief, Department of Oral and Maxillofacial Surgery, Hospital La Luz, Madrid; 6 DMD, DDS, phD. Chief, Department of Oral and Maxillofacial Surgery, Hospital La Luz, Madrid Chief of Section, Department of Oral and Maxillofacial Surgery, Hospital Universitario La Paz, Madrid

## Abstract

A 52-year-old female patient with a diagnostic of osteosarcoma in the mandible, in which it was necessary a reconstruction with a microvascularized osteomyocutaneous fibula bone. Coadjuvant chemotherapy was scheduled. Two years later, 4 osseointegrated implants (OII) were placed in the fibula a 2 OII in the right mandible, using a splint guided surgery. The final prosthodontic consisted in a metal ceramic restoration using CAD/ CAM technology.

** Key words:**Oral rehabilitation, oral cancer, head and neck radiotherapy, oral oncology.

## Introduction

When it comes to implant placement, one of the main problems derives from the effects of radiotherapy on the oral cavity (1), in particular the jaw, which have been extensively described in the literature (2,3). Radiation to the bone provokes endarteritis that leads to hypoxia, hypocellularity, and hypovacularization, accompanied by tissue destruction and a chronic non-regenerable lesion. The extent of the damage will always depend on the radiation dose, type of treatment, and radiation field. Radiotherapy involves a quantitative and qualitative decrease in bone, involving negative factors for bone regeneration (2-4).

The aim of this work was to describe the rehabilitation treatment of a clinical case of oncologic patient with radiation therapy to the head and neck.

## Case Report

**Figure 1:**
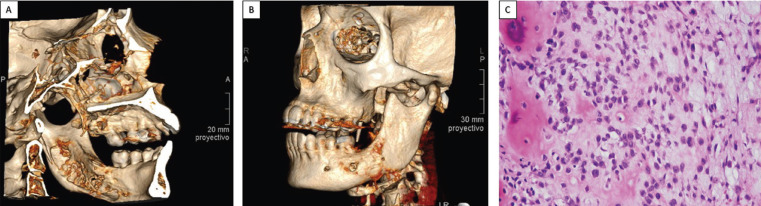
A,B. CT images showing lesion in the left side of the body of the mandible. C. Histological image shows a bone matrix-forming malignant mesenchymal tumor, or osteocarcoma, composed of a dense cellular proliferation in sheet-like formation, with a high nucleus-cytoplasm relation and evident nucleolus. Evidence of mitosis can be seen on the upper edge of the image.

A 52-year-old female patient, diagnosed with hypoesthesia of the inferior alveolar nerve that had evolved over a period of 1 year. This was being monitored by her dentist due to the presence of a periapical lesion on the lower left second molar (3.7), which was assessed at the neurological service at La Paz University Hospital, Madrid, Spain. Three months later, she returned presenting a paramandibular increase in volume on the left side, which did not improve under antibiotic treatment. Exodontia of tooth 3.7 was performed with a biopsy, which revealed an osteosarcoma (Fig. 1).

**Figure 2:**
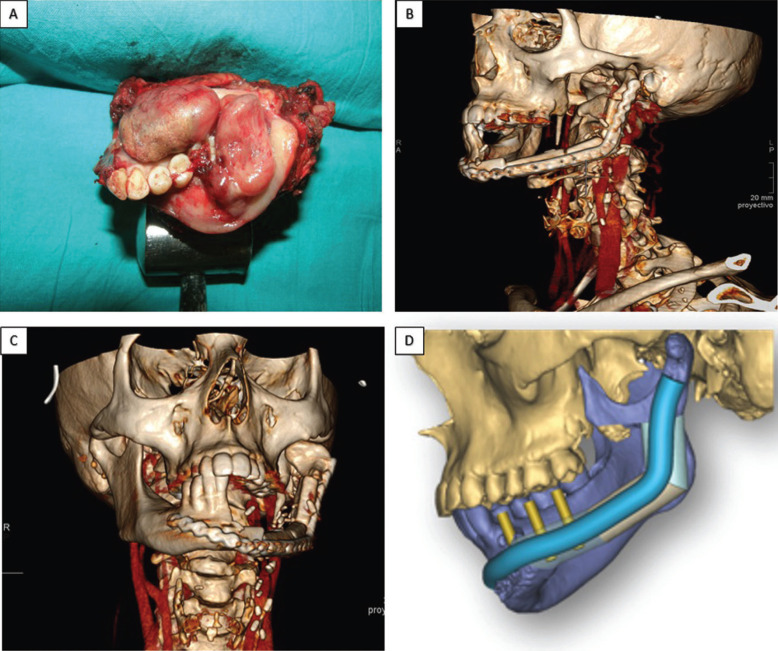
A. Resection piece after excision of the lesion. B,C. CT images captured after reconstruction with microvascularized fibula free flap. D. Planning the surgical reconstruction.

Controlled tracheostomy and intermaxillary blocking were performed, adopting a cervical approach for supramylohyoid cervical dissection with mandibulectomy (under general anesthesia) from the mandibular symphysis to the condyle on the side affected by the lesion, including the soft tissues of the mouth floor, jugal mucosa, and tonsil pillars. During the same surgical session, reconstruction was performed using a buccal fat pad flap and a microvascularized osteomyocutaneous fibula bone graft from the ispsilateral side, with three osteotomies, placing a preformed titanium osteosynthesis plate (Martin®). End-to-end anastamosis to the upper thyroid artery and end-to-side anastomosis to the left jugular vein were performed (Fig. 2A-C). Coadjuvant chemotherapy was scheduled, administering combined cisplatin and adriamycin for six cycles followed by radiotherapy of the affected margins.

Two years later, the patient was found to be free of disease but wished to recover function and esthetics. She was provided with a partial removable prosthesis but this was poorly tolerated due to the unfavorable anatomical situation. It was decided to rehabilitate the patient by means of implants.

**Figure 3:**
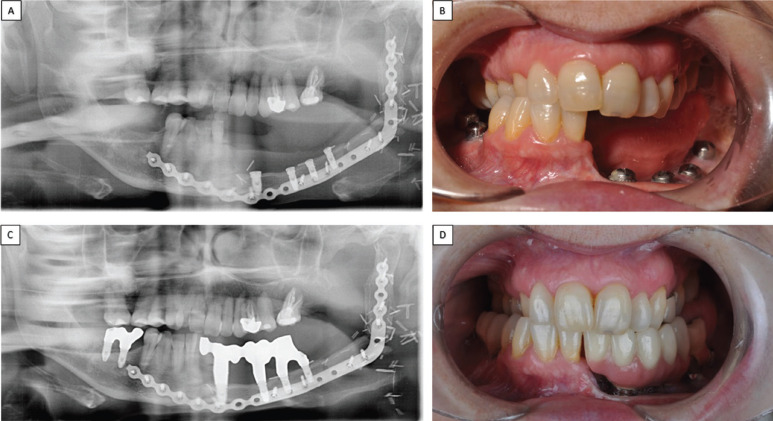
A,B. Orthopantomography and frontal photograph showing OII placement in the fibular free flap region bearing implants with corresponding healing abutments. C,D. Orthopantomography and photograph showing the patient with prosthesis placed in oral cavity.

**Figure 4:**
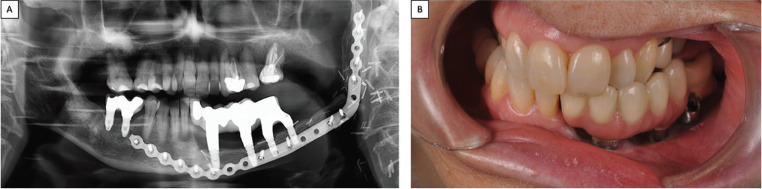
A,B. Ortophantomography and lateral intraoral photograph. Follow –up at 4 years posttreatment.

The presence of the titanium plate with multiple fixing screws was complicated to remove, and so treatment opted for guided implant surgery. To do this, a radiopaque splint was fabricated on a model of the patient processed by means of Materialise 3-D printing software (Fig. 2 D), planning the placement of six implants (4 x 10mm, Biomet 3i Osseotite®) (Fig. 3A,B). After the osteointegration period, two CAD/CAM structures were milled from cobalt chromium (coated with ceramic) fitted to the implant abutments between teeth 31 and 36, and between 46 and 47 (Fig. 3C). The bases of these metal structures were polished where they would be in contact with the mucosa leaving space to allow adequate hygiene maintenance. (Fig. 3D). Four years later, the patient is in good health and the implant rehabilitation has not presented any complications. Periodontal checkups are performed every 6 months (Fig. 4).

## Discussion

In this case, implants were placed two years after surgery and radiotherapy. In the first treatment phase, radiotherapy was considered a contraindication to implant placement. One of the most important factors for implant placement is the effect on the jaw of previous or subsequent radiotherapy. In the soft tissues, radiotherapy provokes inflammation, xerostomy, insertion loss, and increased risk of osteoradionecrosis.1 Numerous studies have assessed the success of dental implants in irradiated cancer patients obtaining disparate results. In 1997, Keller (5) obtained a success rate of 99% in the mandible, while Ryu *et al.* (6) obtained 70%. In a study of osteointegration in patients undergoing radiotherapy, Ali (7) found that only 40% of the implants integrated correctly. But Andersson (8) reported results with a success rate close to 100%. Other authors, such as Jisander (9) and Visch (10) stated that patients subjected to radiotherapy below 50 Gy, present better rates of osteointegration.

In general, most authors evidence better osteointegration when implant placement is delayed for a period after the end of radiotherapy, or when they are placed some time before radiotherapy commences. Jacobsson *et al.* reported that bone regeneration decreased by 70.9% when implants were placed 4 weeks after the end of radiotherapy, but only 28.9% when placement was delayed by 12 months (11). Various animal studies (using animals with equivalent bone metabolism to human bone) have observed bone regeneration in irradiated peri-implant bone providing placement is delayed by 12 months after the completion of radiotherapy (12-14).

Regarding implant survival in irradiated patients, in 2007 Nelson (15), in a study of 93 patients, 29 of them having undergone radiotherapy, obtained a survival rate of 84% 4 years and 54% 13 years after placement respectively, without significant differences between the two groups. In 2012, Buddula (16) published a 20-year retrospective study of 48 patients who received implants in bone previously irradiated with at least 50 Gy, obtaining implant survival rates after 1, 5 and 10 years of 98.9%, 89.9% and 72.3% respectively, which suggests progressive loss of osteointegration as the period of prosthetic loading advances.

Tanakaen, in a systematic review of implants placed in oncologic patients, obtained success rates ranging from 74.4% to 98.9% in both one-piece implants and larger implant-supported prosthetic rehabilitations (17).

In 2014, Schiegnitz conducted an interesting meta-analysis of the influence of radiotherapy on osteointegration. One of its main conclusions was that all the studies of this topic published before 2006 report lower levels of treatment success. After this date, improvements in protocols, implant design and surface treatments, and the use of guided surgery have led to better osteointegration results with statistically significant differences (18).

## Conclusions

Implant based functional rehabilitation is sTable and possible in the long term even in cancer patients undergoing radiotherapy.
