# Correction: Dental implants rehabilitation in a patient with head and neck radiotherapy for osteosarcoma in the jaw. A clinical case report

**DOI:** 10.4317/jced.5122335667797

**Published:** 2021-12-01

**Authors:** Pablo Garrido-Martínez, Juan-Francisco Peña-Cardelles, Norberto Quispe-López, José-Juan Pozo-Kreilinger, Germán Esparza-Gómez, Néstor Montesdeoca-García, José-Luis Cebrián-Carretero

**Affiliations:** 1 DDS, phD. Associate Professor, Department of Prosthesis, Faculty of Dentistry, University Alfonso X el Sabio, Madrid. Department of Oral and Maxillofacial Surgery, Hospital La Luz, Madrid; 2 DDS. Professor of the Postgraduate Program in Oral Surgery and Implantology. Universidad Rey Juan Carlos, Madrid, Spain; 3 DDS, PhD. Associate Professor, Department of Surgery. Faculty of Medicine, Dental clinic. University of Salamanca; 4 MD, DDS, phD. Associate Professor of Medicine. Department of Pathology. Universidad Autónoma de Madrid, Madrid. Hospital Universitario La Paz, Madrid; 5 MD, DDS, phD. Professor Titular, Faculty of Odontology, Universidad Complutense de Madrid, Madrid; 6 DMD, phD. Chief, Department of Oral and Maxillofacial Surgery, Hospital La Luz, Madrid; 7 DMD, DDS, phD. Chief, Department of Oral and Maxillofacial Surgery, Hospital La Luz, Madrid Chief of Section, Department of Oral and Maxillofacial Surgery, Hospital Universitario La Paz, Madrid

In this article by Garrido-Martínez P and colleagues ( J Clin Exp Dent. 2021 Apr 1;13(4):e418-21), there is an error in the authors of the manuscript. The correct author list is: Pablo Garrido-Martínez, Juan-Francisco Peña-Cardelles, Norberto Quispe-López, José-Juan Pozo-Kreilinger, Germán Esparza-Gómez, Néstor Montesdeoca-García, José-Luis Cebrián-Carretero.

